# Supplementary material for: Shared Treg dysregulation underlies psoriasis vulgaris and atopic dermatitis through the PHF19-PRC2 epigenetic axis
Source: Front Immunol. 2026 Aug 20;17:1932788. doi: 10.3389/fimmu.2026.1932788 (PMC13538097; doi:10.3389/fimmu.2026.1932788)
Supplement: Supplementary file 3 [file DataSheet3.zip › Additional files/Additional file 2/52.BTN3A2.forest.pdf]

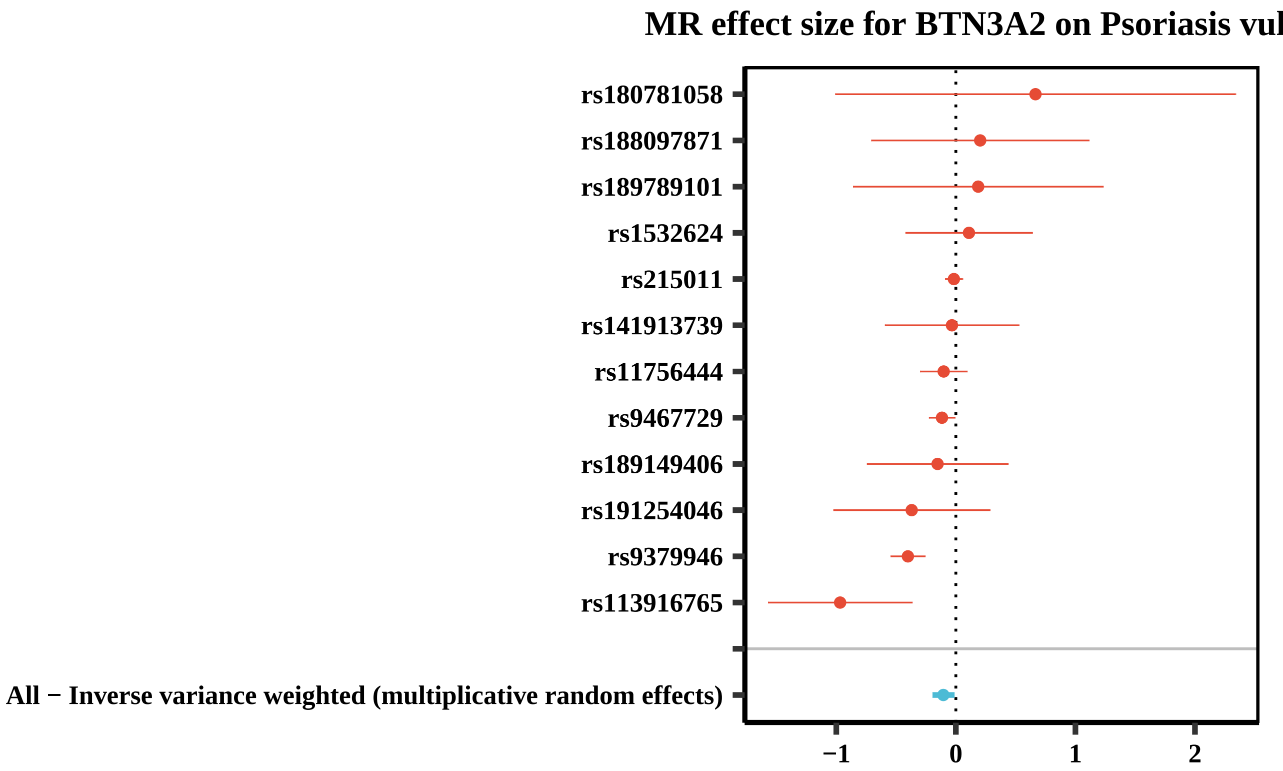
MR effect size for BTN3A2 on Psoriasis vul
rs180781058
rs188097871
rs189789101
rs1532624
rs215011
rs141913739
rs11756444
rs9467729
rs189149406
rs191254046
rs9379946
rs113916765
All − Inverse variance weighted (multiplicative random effects)
−1
0
1
2
